# Lactose Malabsorption and Presumed Related Disorders: A Review of Current Evidence

**DOI:** 10.3390/nu14030584

**Published:** 2022-01-28

**Authors:** Paolo Usai-Satta, Mariantonia Lai, Francesco Oppia

**Affiliations:** 1 Gastroenterology Unit, Brotzu Hospital, 09121 Cagliari, Italy; f.oppia@tiscali.it; 2 Gastroenterology Unit, University of Cagliari, 09042 Monserrato, Italy; toninalai@medicina.unica.it

**Keywords:** lactose malabsorption and intolerance, celiac disease, inflammatory bowel disease, irritable bowel syndrome, bone mass density, Hashimoto’s thyroiditis

## Abstract

Background. Lactose malabsorption (LM) is a frequent clinical problem associated with several digestive and extra-digestive diseases. The aim of this manuscript was to clarify the real clinical impact of LM on these disorders. Methods. A literature search for digestive and extra-digestive disorders related to LM was carried out using PubMed, Medline and Cochrane. Results. A transient lactase deficiency is present in celiac disease (CD) on a normal diet. The persistence of symptoms in CD on a gluten-free diet may be instead, in part, attributed to a primary LM. Similar circumstances are present in inflammatory bowel diseases (IBD), in which LM can be responsible for a part of persistent symptoms in IBD on clinical remission. LM and irritable bowel syndrome (IBS) are instead independent conditions. On the other hand, a lactose-restricted diet may be useful for some IBS patients. A reduced lactose intake can lead to low bone mass and limited risk of fragility fractures. Finally, the absorption of levothyroxine could be conditioned by LM. Conclusions. LM can be responsible for persistent symptoms in CD and IBD. The association with IBS seems to be casual. Bone mass and levothyroxine absorption can be affected by LM.

## 1. Introduction

Lactose malabsorption (LM) is a frequent clinical condition caused by lactase-reduced activity, which may be responsible for digestive symptoms such as abdominal pain, bloating and diarrhoea, both in adults and in children, namely lactose intolerance (LI) [1,2,3,4]. As a consequence of an osmotic action that increases the volume of intestinal contents, the undigested lactose is fermented by the colonic flora, exacerbating digestive symptoms. Non-invasive hydrogen breath testing (BT) is generally considered the most reliable and recommended diagnostic technique [5,6] to detect LM. On the other hand, LI is not synonymous with LM. In fact, only some patients with LM report symptoms. A blind lactose challenge should be the recommended method to objectively demonstrate LI [5]. A lactose-restricted diet and the use of pharmacological lactase supplements represent the recommended therapeutic strategy.

LM and LI have been associated with various digestive and extra-digestive problems, such as celiac disease (CD), inflammatory bowel disease (IBD), irritable bowel syndrome (IBS) and also low bone mass and hypothyroidism [3]. The spectrum of disorders related to LM has emerged as a potentially relevant argument possibly related to health care management [4], although controversial data are available in this regard. The aim of this paper was to clarify the clinical impact and the real relationship of LM with the most common related disorders.

## 2. Methods

A review of the literature on LM/LI related to digestive and extra-digestive diseases was conducted using the online databases of PubMed, Medline and Cochrane. The most relevant original research, review, systematic review, meta-analysis and eventually books were included in the search and performed in various combinations with the Boolean operators ‘and’, ‘or’, and ‘not’, selecting articles published in English.

## 3. Lactose Malabsorption and Intolerance

### 3.1. Background

Lactose is a disaccharide composed of glucose and galactose present in milk and dairy products. Absorption of lactose requires lactase-phlorizin hydrolase (LPH) activity in the small intestinal brush border. LM is directly caused by LPH deficiency, namely hypolactasia [2,3,4,5,6]. Hypolactasia comprises three distinct forms: congenital, primary and secondary [3,5].

Congenital hypolactasia is an extremely rare lifelong disorder characterised by difficulty in growth and severe diarrhoea from the first exposure to milk. Congenital lactase deficiency is caused by a single autosomal recessive disorder [7].

Primary hypolactasia occurs in a large proportion of the population, and it is the most frequent condition related to LM [5]. In particular, primary lactase deficiency is related to an autosomal recessive condition resulting from the physiological and progressive decline of lactase activity in the intestinal brush border [5]. A single nucleotide polymorphism, C/T-13910, has been correlated with lactase persistence or not persistence in several populations [5,8]. The prevalence of primary hypolactasia (lactase non-persistence) varies across the world, with defined lower prevalence in Northern Europe (<5%), compared with Southern Europe (70–90%) and Southeast Asia (almost 100%). [5] The onset of primary hypolactasia is correlated to age: lactase activity is highest at birth and declines after weaning up to 8–12 years of age [9].

Finally, secondary hypolactasia is associated with several diseases such as acute gastroenteritis, celiac disease and Crohn’s disease, which may lead to a transient lactase deficiency [3,5].

Diarrhoea, bloating and abdominal pain are the most common symptoms related to LM. The presence of intolerance symptoms in LM patients confirms a condition of LI. [3,5]. On the other hand, hypolactasia does not necessarily result in the development of digestive disturbances [10]. The presence of LI after lactose ingestion is proportional to the amount of lactose, foods co-ingested with lactose, the lactose fermentation by the colonic flora and visceral sensitivity [3,4,5].

### 3.2. Diagnosis and Management of LM/LI

Lactase assay after jejunal biopsy represents the classic diagnostic gold standard for diagnosis of LM [2,3,4]. However, this diagnostic approach is too invasive for the diagnosis, and its results may be affected by the irregular dissemination of lactase activity throughout the small bowel mucosa [2,3,4,5].

For this reason, lactose BT is commonly considered the most simple, reliable and inexpensive diagnostic instrument to detect LM [5,6,11,12,13].

This non-invasive technique showed good sensitivity (mean value of 77.5%) and excellent specificity (mean value of 97.6%) [14].

A genetic test based on C/T-13910 polymorphism, associated with lactase persistence/not persistence, has recently enriched the diagnostic armamentary of the gastroenterologist [15]. Lactose BT and the genetic test showed an excellent diagnostic correlation [16,17]. In addition, the genetic test may complement BT in subjects with false negative lactose BT, in the paediatric population and in secondary forms of LM [15,16].

Following the lactose challenge, the presence of abdominal pain, bloating, flatulence and diarrhoea may be recorded during and after the BT. This evaluation has been proposed as a simple diagnostic method to demonstrate LI in clinical practice [13,14]. On the other hand, only a part of patients with positive BT report symptoms during and after BT and, conversely, several patients with negative BT diagnose themselves as being lactose intolerant [10].

Although difficult to perform in clinical practice, only a blind lactose challenge should be the recommended method to objectively demonstrate LI [5,18,19].

Finally, in the presence of LI, a lactose-restricted diet, the use of milk and dairy products in which the lactose has been pre-hydrolysed, and pharmacological lactase supplements represent the recommended therapeutic strategy avoiding a total exclusion of lactose by the diet for the risk of the nutritional disadvantages of reduced calcium and vitamin intake [5,18,19].

## 4. Lactose Malabsorption and Celiac Disease

Patients with CD with substantial intestinal damage can experience secondary LM and LI due to decreased lactase activity in the enterocyte’s brush border [20]. Generally, this lactase deficiency is transient, and after gluten withdrawal, normal lactose absorption is restored. When assessed in children, secondary LM was found, ranging from 10 to 19% of celiac patients [21,22]. In 2005, Ojetti et al. [23] suggested serologically screening every positive lactose BT patient for CD, considering LM/LI patients at high risk of CD, compared with healthy subjects. Unfortunately, no further studies are available to confirm these data and to definitely recommend a serological screening of CD in positive lactose BT patients.

After the administration of a gluten-free diet (GFD), up to 30% of celiac patients continue to experience digestive symptoms [24]. These subjects are classified as having non-responsive CD.

Non-responsive CD may be associated with poor adherence to a GFD or refractory CD or other digestive disorders alternative to CD despite a strict GFD and a resolution of villous atrophy [25].

In this last condition, primary LM can be present at the time of CD diagnosis and manifest after gluten withdrawal with new or persistent digestive symptoms.

An LM/LI could account for 8–10% of global non-responsive CD cases [26]. This association is obviously proportionate to the worldwide epidemiological distribution of primary LM.

In any case, the prevalence of primary hypolactasia in CD patients did not differ from controls in a prospective study using a lactase genetic test [27].

After a diagnostic confirmation of LM in CD patients on GFD, the therapeutic management consists of a lactose-restricted diet and the use of milk and dairy products in which the lactose has been pre-hydrolysed [5,18,19].

## 5. Lactose Malabsorption and Inflammatory Bowel Diseases

IBD comprises mainly Crohn’s disease and ulcerative colitis. A meta-analysis by Szilagyi et al. [28] showed an increased risk of LM only in Crohn’s disease with small bowel involvement. This can be due to a secondary and probably transient form of LM/LI [29]. In addition, the outcome of the meta-analysis suggests that hypolactasic (primary LM) and normolactasic persons may be equally affected by IBD. According to several studies, the prevalence of primary LM in IBD patients does not differ from that of controls [30].

On the other hand, an overall prevalence of 35% of patients with IBD experiences digestive symptoms despite clinical remission [30]. Apart from irritable bowel disease (IBS), LM can account in part for persistent digestive symptoms in IBD patients on clinical remission. Notably, the association between IBD and primary LM depends on the epidemiological population distribution of lactase persistence or non-persistence [8]. In this respect, a recent study [31] showed an increased risk of LI in IBD patients compared with controls.

The meta-analysis by Szilagyi [28] also evaluated dairy food effects on IBD. Dairy foods could decrease the risks of IBD, and dairy restrictions could adversely affect disease outcomes.

## 6. Lactose Malabsorption and Irritable Bowel Disease

IBS is the most frequent functional gastrointestinal disorder causing abdominal pain, bloating and altered bowel habits [32]. This condition accounts for 10–15% of the general population and is associated with a decreased quality of life (QoL). The relationship between LM and IBS is controversial. The symptoms of lactose intolerance are often difficult to distinguish from those of IBS, although patients who are lactose intolerant should develop symptoms only after ingesting lactose-containing products. On the other hand, the prevalence of LM in IBS seems to be similar to that observed in the general population [33,34]. Varjú et al. [35] performed a meta-analysis with a systematic review and found that LI, but not LM, was more frequent among patients with IBS compared with healthy controls. In 2001, Vernia et al. [36] showed that the prevalence of a positive lactose BT was similar in patients with IBS compared with patients with suspected milk intolerance. These authors suggested including BT in the diagnostic work-up of IBS. On the contrary, hydrogen BT for LM should have no role in the routine assessment of suspected IBS, according to a recent review [37].

In addition, the impact of a lactose-restricted diet in patients with IBS is debated. Lactose is a component of the FODMAPs (highly fermentable oligo-, di-, monosaccharides and polyols) diet. Several studies confirmed the positive impact of a low FODMAP diet (LFD) in improving digestive symptoms in IBS [38,39,40]. On the other hand, a lactose BT should be performed before an LFD in populations with a low prevalence of LM, avoiding an inappropriate exclusion of lactose in lactase-persistent IBS patients [5]. Apart from FODMAPs, a lactose-restricted diet should be reserved for hypolactasic IBS patients. In conclusion, a restriction of lactose should be not systematically recommended in patients with IBS.

## 7. Lactose Malabsorption and Bone Mass

An adequate calcium intake is essential to building and maintaining bone. Dairy products provide the most concentrated sources of calcium in the diet [41]. LM and a lactose-restricted diet may predispose individuals to low calcium intake. However, the majority of studies showed that neither dietary lactose nor hypolactasia has a significant direct impact on calcium absorption in healthy adults [42]. Otherwise, many observational studies showed that children with lactose-free or low lactose diets had reduced bone mass density (BMD). A meta-analysis [43] about dietary calcium in children showed a significantly higher total body and lumbar spine BMD in groups with higher compared with low calcium intakes. In adults and in the elderly population, a lactose-restricted diet can have a negative impact on BMD, according to several studies [44,45,46].

On the other hand, whether low dairy consumption also leads to higher risks of bone fracture is still controversial. A meta-analysis of 12 studies [47] reported no overall association between milk intake and hip fractures. On the contrary, a recent study [48] that included 123,906 elderly men and women followed for up to 32 years showed that each additional serving of dairy products per day was associated with an 8% lower risk of hip fracture. Despite the unequivocal evidence, a decreased intake of dairy products could lead to a relatively low but significant risk of fragility fractures.

In any case, public health organisations recommend that all individuals, including patients with LI, consume three servings of dairy per day to ensure optimal bone health [49].

## 8. Lactose Malabsorption and Thyroid

As a consequence of LM, undigested lactose draws water into the intestinal lumen and leads to bacterial fermentation. This causes osmosis and accelerates small intestinal transit, which could lead to insufficient L-thyroxine absorption in hypothyroid patients [50].

In 2014, Asik et al. [51] found LM in 75% of patients with Hashimoto’s thyroiditis. In addition, they observed decreased levels of thyroid-stimulating hormone (TSH) after lactose restriction. On the other hand, LI was diagnosed by a no validated test (lactose load test), and no data were available about the prevalence of LM/LI in the related general population.

Another study by Cellini et al. [52] showed that LM significantly increased the need for oral levothyroxine in hypothyroid patients.

Therefore, LI could reduce the bioavailability of this drug and enforce the use of higher doses, although further studies are needed to confirm these data.

## 9. Conclusions

Table 1 summarises the prevalence and the clinical impact of LM/LI on the most common related disorders. The prevalence of LM/LI in CD and IBD patients does not differ from that observed in the general population. LI may be responsible for persistent symptoms in CD and IBD patients on clinical remission. IBS and LM are casually associated, although a lactose-restricted diet (within a low FODMAP diet) can be useful in some IBS patients.

Although LM does not have a direct impact on calcium absorption, a dairy-restricted diet can have a negative impact on BMD and lead to a limited risk of fragility fractures.

Finally, LM and LI could reduce the bioavailability of levothyroxine in hypothyroid patients and enforce the use of higher doses.

In any case, further studies are needed to confirm or reinforce this evidence.

## Figures and Tables

**Table 1:** Lactose malabsorption and the most common related disorders: prevalence and clinical impact.

	Prevalence	Clinical Impact
CD	Not increased (primary hypolactasia)	Secondary transient hypolactasia Persistent symptoms on gluten-free diet
IBD	Not increased (primary hypolactasia)	Secondary transient hypolactasia (Crohn’s disease) Persistent symptoms on clinical remission
IBS	Not increased	Lactose-restricted diet useful in hypolactasic IBS
Low bone mass	Not defined	Dairy-restricted diet can cause low bone mass and hip fractures
Hypothyroidism	Not defined	Reduced levothyroxine absorption

Notes: CD, celiac disease; IBD, inflammatory bowel disease; IBS, irritable bowel syndrome.

## Data Availability

Not applicable.
